# Draft Genome Sequence of Multidrug-Resistant Vibrio parahaemolyticus Strain PH698, Infecting Penaeid Shrimp in the Philippines

**DOI:** 10.1128/MRA.01040-19

**Published:** 2019-11-21

**Authors:** Cynthia P. Saloma, Sarah Mae U. Penir, Joseph Matthew R. Azanza, Leobert D. dela Pena, Roselyn C. Usero, Nikko Alvin R. Cabillon, Angela Denise P. Bilbao, Edgar C. Amar

**Affiliations:** a National Institute of Molecular Biology and Biotechnology, University of the Philippines, Diliman, Quezon City, Philippines; b Philippine Genome Center, University of the Philippines, Diliman, Quezon City, Philippines; c Southeast Asian Fisheries Development Center, Aquaculture Department, Tigbauan, Iloilo, Philippines; d Negros Prawn Producers Cooperative (NPPC), Bacolod City, Negros Occidental, Philippines; Georgia Institute of Technology

## Abstract

The emergence of multidrug-resistant bacterial strains in diverse settings has been reported globally. In the Philippine shrimp aquaculture industry, antibiotics are used for the treatment of bacterial diseases during the production cycle. We report the draft genome of Vibrio parahaemolyticus PH698, a multidrug-resistant strain isolated from a Philippine shrimp farm.

## ANNOUNCEMENT

Antibiotics are commonly used by aquaculture farmers to prevent and treat bacterial infections in shrimp (1). The range of commonly used antibiotics varies widely between shrimp-producing countries due to different management and regulation practices. However, on account of extensive antibiotic use, there is an observable trend of the emergence of antibiotic-resistant strains in shrimp farms (2, 3). In the Philippines, there is no existing report of a multidrug-resistant Vibrio parahaemolyticus strain isolated from shrimp. Here, we describe the draft genome sequence of V. parahaemolyticus PH698, a multidrug-resistant strain isolated from the hepatopancreas of Penaeus vannamei shrimp in a pond with an outbreak of unknown etiology.

Shrimp samples exhibiting clinical signs of disease were preferentially collected for dissection and homogenization of their hepatopancreas. Serial dilutions of the hepatopancreas homogenate in sterile seawater were plated in nutrient agar (Pronadisa) with 2% NaCl. The colony of Vibrio parahaemolyticus strain PH698 was isolated to generate pure bacterial cultures. The isolated strain was grown overnight in nutrient broth (Pronadisa) with 1.5% NaCl at 30°C. Automated genomic DNA extraction was performed with the KingFisher cell and tissue DNA kit (Thermo Fisher Scientific). A paired-end library was prepared from the genomic DNA using the Nextera XT library preparation kit (Illumina) and was sequenced on the Illumina MiSeq platform (2 × 300 bp, MiSeq reagent kit v. 3) at the DNA Sequencing Core Facility, Philippine Genome Center, at a coverage of >80×.

The quality of the reads was checked and verified using FastQC (4). Adapter sequences were removed from 2,662,434 paired-end reads using Cutadapt v. 1.18 (5). The trimmed paired-end reads were assembled into 62 contigs (*N*_50_, 193,506 bp) longer than 1,000 bp using SPAdes v. 3.13.0 (6) with k-mer sizes of 21, 33, 55, 77, 99, and 127 with mismatch correction. The largest contig has a length of 468,564 bp, and the total assembly size is 5,342,783 bp. The G+C content of the draft genome sequence of the strain is 45.5%. The assignment of strain PH698 as V. parahaemolyticus was established when the average nucleotide identity (ANI) values between the DNA sequence of the isolate and other publicly available strains of the species were validated to be >95% using the aniM option of calculate_ani.py (7). Annotation of the draft genome with the NCBI Prokaryotic Genome Annotation Pipeline (PGAP) v. 4.9 (8) led to the identification of 5,084 coding sequences (CDSs), 104 tRNAs, 13 rRNAs, and 4 noncoding RNAs (ncRNAs).

Detection of antibiotic resistance genes from the sequences in the Antibiotic Resistance Gene-ANNOTation (ARG-ANNOT) database (9) in the raw reads of the strain was executed using the default parameters of SRST2 v. 0.20 (10). V. parahaemolyticus strain PH698 contains 8 antibiotic resistance genes, namely, *aac3iv*, *ant3ia*, *dfra17*, *erea*, *aph33ib*, *aph6id*, *sul2*, and *tetc*. Altogether, the genes confer resistance for different antibiotic classes, ranging from aminoglycosides (e.g., apramycin, dibekacin, gentamicin, netilmicin, sisomicin, spectinomycin, streptomycin, and tobramycin), trimethoprim, macrolide-lincosamide-streptogramin (e.g., erythromycin), sulfonamides, and tetracyclines.

### Data availability.

The draft genome sequence and annotation of V. parahaemolyticus PH698 have been deposited in GenBank under accession no. VSBR00000000 and assembly accession no. GCF_008271865. Illumina reads have been deposited in the SRA under accession no. SRR9870108.
